# Case Report: Ribociclib-associated bilateral neurotrophic keratitis requiring amniotic membrane transplantation during adjuvant treatment of early breast cancer

**DOI:** 10.3389/fonc.2026.1932226

**Published:** 2026-10-09

**Authors:** Danielle DeCicco, Sanjana Nethagani, Lingo Lai, Danish Safi

**Affiliations:** 1 Department of Medical Oncology, West Virginia University Cancer Institute, Morgantown, WV, United States; 2 Department of Ophthalmology and Visual Sciences, West Virginia Eye Institute, Morgantown, WV, United States

**Keywords:** amniotic membrane transplantation, breast cancer, case report, CDK 4/6 inhibitor, neurotrophic keratitis, ocular adverse drug event, pharmacovigilance, ribociclib

## Abstract

**Background:**

Cyclin-dependent kinase (CDK) 4/6 inhibitors with endocrine therapy are standard of care for hormone receptor (HR)-positive, HER2-negative breast cancer in metastatic and adjuvant settings. The ribociclib label lists dry eye and increased lacrimation among ocular reactions in under 10% of patients, and isolated vortex keratopathy, epithelial keratopathy, and palinopsia are described. To our knowledge, neither neurotrophic keratitis (NK) nor management of an ocular adverse event with amniotic membrane transplantation (AMT) has been reported with any CDK4/6 inhibitor.

**Case:**

A 42-year-old premenopausal woman with stage IIB (cT2N1) HR-positive/HER2-negative invasive ductal carcinoma achieved a pathologic complete response after neoadjuvant chemotherapy and bilateral mastectomy. Adjuvant abemaciclib was discontinued after one month for refractory gastrointestinal toxicity, without ocular complaints. Ribociclib 400 mg/day was initiated. After approximately four months she developed bilateral blurred vision; optometry identified bilateral inferior superficial punctate keratitis (SPK), and preservative-free lubrication was started. At the fifth-month visit, bilaterally reduced corneal sensitivity, a feature of early neurotrophic keratopathy, was documented, and prednisolone acetate 1% was begun. With symptoms failing to improve, ribociclib was held. At the six-month visit, with central SPK, tear-film instability, and further visual decline despite corticosteroid, amniotic membrane grafts with bandage contact lenses were placed sequentially in each eye, and topical cyclosporine 0.05% was started. Ribociclib was permanently discontinued. Right-eye vision normalized within five months; the left eye recovered over fifteen months. Anastrozole and all other concomitant medications were continued without recurrence.

**Conclusion:**

To our knowledge, this appears to be the first reported case of bilateral neurotrophic keratitis during CDK4/6 inhibitor therapy and the first ribociclib-associated ocular adverse event refractory to standard topical therapy that improved after procedural intervention. A Naranjo score of 5 indicates a probable association, supported by a documented normal ocular baseline on the same concomitant medications, absence of ocular toxicity during brief prior abemaciclib exposure, and improvement after dechallenge with continued anastrozole. As a single, signal-generating case, causality cannot be established; additional reports and pharmacovigilance analyses are needed. As adjuvant use expands, clinicians should watch for corneal nerve toxicity beyond dry eye, ask routinely about visual symptoms, and refer early for eye care.

## 1 Introduction

Cyclin-dependent kinase (CDK) 4/6 inhibitors—palbociclib, ribociclib, and abemaciclib—have become cornerstones in the treatment of HR-positive/HER2-negative breast cancer (1). Ribociclib is an orally administered, selective small-molecule inhibitor of CDK4 and CDK6 that blocks cyclin D–CDK4/6-mediated phosphorylation of the retinoblastoma protein and arrests G1–S cell-cycle progression (2, 3). As a lipophilic weak base, it distributes widely into tissues and accumulates within acidic organelles such as lysosomes, a physicochemical property relevant to its proposed ocular effects (4). Ribociclib received FDA approval in September 2024 for adjuvant treatment of stage II and III high-risk early breast cancer based on the NATALEE trial, which demonstrated a significant invasive disease-free survival benefit (HR 0.749; 95% CI 0.628–0.892) and is expected to substantially expand the population exposed to long-duration adjuvant therapy (1, 2).

The well-characterized adverse event profile of ribociclib emphasizes neutropenia, hepatotoxicity, QT prolongation, and gastrointestinal toxicity (2, 3). Ocular adverse events have received comparatively little attention. The label cites dry eye (4–5%) and increased lacrimation (4%) as the principal corneal-surface findings observed in the MONALEESA program (3, 5). A recent disproportionality analysis of the FDA Adverse Event Reporting System (FAERS) identified 439 ocular adverse event reports for ribociclib, with a significantly higher ocular toxicity than abemaciclib on multivariate analysis (6). Beyond label reactions, isolated published reports include vortex keratopathy (7–9), bilateral epithelial keratopathy with punctate keratitis (10), and dose-related palinopsia (11).

Neurotrophic keratopathy (NK) is a degenerative corneal disease driven by impaired trigeminal innervation, characterized by reduced corneal sensation, tear-film instability, and progressive epithelial breakdown that can culminate in stromal ulceration or perforation (12, 13). Common etiologies are herpetic infection, surgical nerve injury, diabetes, and drug toxicity (14). To our knowledge, NK has not previously been reported with any CDK4/6 inhibitor, and the corneal findings described in earlier reports of ribociclib were consistent with epithelial or lysosomal injury rather than denervation.

We describe a patient who developed bilateral neurotrophic keratitis with reduced corneal sensitivity during adjuvant ribociclib therapy, refractory to standard topical therapy, requiring bilateral amniotic membrane placement with bandage contact lenses and permanent discontinuation of the drug. Within the scope of the literature search described in Section 3.3, the case appears to extend the ocular toxicity profile of ribociclib in three respects:

- To our knowledge, it is the first report of clinically assessed neurotrophic features (reduced corneal sensation on qualitative testing) in a patient treated with a CDK4/6 inhibitor.
- It appears to be the first published ribociclib-associated ocular adverse event refractory to standard topical corticosteroid and lubrication that was of sufficient severity to be managed with a procedural intervention (amniotic membrane placement).
- It highlights the clinical importance of timely attention to non-resolving ocular symptoms during CDK4/6 inhibitor therapy, with prompt response and cessation of the drug, plausibly materially shaping the outcome.

## 2 Case description

### 2.1 Patient and oncologic history

A 42-year-old premenopausal woman with no prior ocular history presented with a self-palpated left breast mass. Her last comprehensive eye examination, three years before ribociclib initiation, was normal. It was specifically negative for tear-film insufficiency, corneal pathology, glaucoma, cataract, and macular disease. She wore corrective lenses only for myopia and did not wear contact lenses. Medical history included asthma, gastroesophageal reflux, and anxiety/depression; she was a current smoker. Relevant to neurotrophic keratopathy risk, the documented history denied diabetes mellitus and hypertension, and the ocular history denied glaucoma, tear-film insufficiency, cataract, degenerative macular disease, injury of the eye region, diabetic retinopathy, vision impairment, strabismus, and amblyopia. No autoimmune or neurologic disease was recorded on review of systems. Family history was notable for breast cancer in a maternal aunt (PALB2 heterozygote).

Bilateral diagnostic mammography revealed a left BI-RADS (Breast Imaging Reporting and Data System) 5 lesion, highly suggestive of malignancy, and a right BI-RADS 0 finding. Left breast ultrasound demonstrated multiple masses (largest 23 × 16 mm) in the upper outer quadrant. Core biopsy confirmed invasive ductal carcinoma with medullary features, Nottingham grade 3, ER 95%, PR 60%, HER2 2+ (FISH negative), Ki-67 60%, with lymphovascular invasion. A 7 mm morphologically abnormal axillary node was biopsy-positive. PET-CT showed no distant metastasis. A 40-gene panel was negative for pathogenic mutations. Clinical stage was IIB (cT2N1).

### 2.2 Treatment course

Neoadjuvant therapy comprised dose-dense doxorubicin/cyclophosphamide (ddAC) × 4 cycles followed by weekly paclitaxel × 4 cycles (15, 16). The patient subsequently underwent left total mastectomy with axillary lymph node dissection (ALND) and right prophylactic mastectomy; pathology demonstrated a pathologic complete response (ypT0, pN0(i+); 2/12 nodes with isolated tumor cells, negative margins). Adjuvant radiation therapy (25 fractions) was completed approximately four months postoperatively.

A GnRH (gonadotropin-releasing hormone) agonist was initiated, and baseline DEXA showed osteopenia. Adjuvant abemaciclib was started with anastrozole 1 mg daily per the monarchE protocol (17, 18), but was discontinued after roughly one month for refractory diarrhea and fatigue persisting through dose reduction. No ocular complaints were recorded during abemaciclib exposure. Bilateral salpingo-oophorectomy was then performed, and the GnRH agonist was stopped. After informed consent and a baseline electrocardiogram, ribociclib 400 mg/day (200 mg × 2; 21 days on/7 days off) was initiated, together with anastrozole 1 mg daily. The 400 mg dose was used throughout her ribociclib exposure; no dose reductions were performed.

### 2.3 Onset and progression of ocular adverse event

At the three-month oncology review on ribociclib, there were no reported ocular symptoms. The patient was tolerating ribociclib, with a stable QTc and laboratory findings of mild leukopenia (WBC 2.9 × 10^3^/µL) and lymphopenia (0.71 × 10^3^/µL). Serial ocular findings at each subsequent visit are summarized in **Table 1**.

**Table 1:** Standardized serial ocular findings by visit.

Visit	BCVA (OD; OS)	Refraction/IOP	SPK (grade; distribution)	Corneal sensitivity	TBUT	Intervention initiated
Month −37 (baseline)	Normal with correction	Myopic correction; IOP normal	None; no ocular surface disease	NR (no complaints)	NR	None
Month 4	20/20−; 20/20	OD −4.25 −0.25 ×095; OS −4.50 −0.75 ×090/20; 22 mmHg	1+ OD, 2+ OS; inferior	NR	NR	PF artificial tears (Refresh Digital PF) QID
Early Month 5	20/25 + 2; 20/30	OD −5.00 −0.25 ×095; OS −5.00 −0.50 ×090; +0.50 add/NR	1+ OD, 2+ OS; central (migrated from inferior)	3+ central OU; 1+ superior OS; 0 all other quadrants	6 s OU	Prednisolone acetate 1% QID OU; tears QID continued
Month 6 (AMT visit)	Further reduced [values NR]*	Refractive instability/21; 21 mmHg	1+ OD, 2+ OS; central (stable)	Previously reduced OU (per note)	6 s OU (stable)	Staged AMT (Apollo 12 mm) + BCL (−0.25 DS): OS this visit, OD at 1-week recheck; cyclosporine 0.05% BID; prednisolone stopped; PF tears QID continued
Month 6 + 1 wk (recheck)	Improved OU [values NR]*	NR	1+ central OD (untreated); none noticeable OS (no stain instilled)	NR	NR	OD AMT (Apollo 12 mm) + BCL (−0.25 DS); tears QID + cyclosporine BID continued
Month 6 + 2 wk (recheck)	20/25; 20/30	NR	Trace OD (single pinpoint); 1+ inferior band OS (partial recurrence after membrane/BCL removal)	NR	NR	BCLs removed; nightly lubricating gel added OU; RTC 3 mo; cenegermin contingency documented
Month 12	OD normalized; OS improving*	Stabilizing/NR	Resolved OD; improving OS	NR	NR	Cyclosporine continued
Month 16	Improved OU; OS mildly symptomatic*	Stable/NR	Improving	NR	NR	Cyclosporine continued
Month 22	Improved OU*	Stable/19; 19 mmHg	None OD (clear, no staining); trace OS	Noted as normal	OD >10 s; OS 7 s	Cyclosporine BID + PF tears QID continued; ergonomic advice for computer use

BCVA, best-corrected visual acuity; IOP, intraocular pressure; SPK, superficial punctate keratitis; TBUT, tear break-up time; AMT, amniotic membrane transplantation; BCL, bandage contact lens; PF, preservative-free; NR, not recorded; OD, right eye; OS, left eye; OU, both eyes; QID, four times daily; BID, twice daily; RTC, return to clinic. Corneal sensitivity was assessed clinically (qualitative cotton-wisp response, graded 0 [absent] to 3+ [brisk/intact]); quantitative esthesiometry was not performed.

*Formal Snellen best-corrected visual acuity was not recorded at this visit; the entry reflects the clinician’s documented qualitative assessment.

Approximately one month later (four months after being on ribociclib), she presented to optometry with bilateral blurred vision at distance and near, mild and constant throughout the day. Initial symptoms had been gradual; she had attributed them to the need for an updated prescription for spectacles. Examination showed best-corrected visual acuity (BCVA) 20/20− in the right eye and 20/20 in the left eye with a new manifest refraction of −4.25 −0.25 ×095 in the right eye and −4.50 −0.75 ×090 in the left eye. Intraocular pressure (IOP) was 20 mmHg in the right eye and 22 mmHg in the left eye by non-contact tonometry, and bilateral inferior superficial punctate keratitis (SPK) graded 1+ and 2+ were noted in the right eye and left eye, respectively. Fundus photography showed a normal posterior segment exam bilaterally. Diagnoses recorded were: bilateral punctate keratitis, bilateral myopia, and bilateral regular astigmatism. Preservative-free artificial tears were recommended four times daily. No epithelial defect or ulcer was present.

At the second optometry visit one month later (early Month 5), corneal sensitivity was formally graded by quadrant: 3+ centrally in each eye, 1+ superiorly in the left eye, and 0 in all remaining quadrants. It was reduced in all quadrants except centrally in the right eye and except centrally and superiorly in the left eye, with the left superior quadrant itself greatly reduced with cotton wisp test. The SPK had migrated from an inferior to a central distribution (1+ right eye, 2+ left eye), and tear break-up time (TBUT) was 6 seconds in each eye. BCVA was 20/25 + 2 in the right eye and 20/30 in the left eye, and manifest refraction had shifted to −5.00 −0.25 ×095 in the right eye and −5.00 −0.50 ×090 in the left eye (right-eye sphere −4.25 → −5.00 over 30 days), with a new +0.50 add required and anti-fatigue lenses recommended. The assessment was early neurotrophic keratopathy consistent with Mackie stage 1. Prescribed prednisolone acetate 1% (Pred Forte) four times daily in both eyes, with continued preservative-free artificial tears four times daily. A two-week recheck was planned to assess corticosteroid response, with a documented next-line plan of amniotic membrane placement and cyclosporine 0.05% twice daily if no response was observed. Quantitative Cochet-Bonnet esthesiometry was not performed.

Between these visits (mid-Month 5), because ocular symptoms did not improve with corticosteroid therapy and lubrication, ribociclib was held pending a repeat eye evaluation.

At the six-month optometry visit, after three weeks of prednisolone, the examination was essentially unchanged: 1+ central SPK in the right eye, 2+ central SPK in the left eye, TBUT stable at 6 seconds in each eye, and intraocular pressure of 21 mmHg bilaterally. Visual acuity had declined further. The optometrist assessed bilateral superficial punctate keratitis, worse in the left eye, with no improvement on corticosteroid therapy, judged “possibly early neurotrophic” given the previously documented quadrant-graded reduction in corneal sensitivity. No epithelial defect or ulcer was recorded. Clinically, it was assessed and treated as neurotrophic keratitis. Treatment was escalated: a dehydrated amniotic membrane graft (Apollo Amniotic Allograft, 12 mm) was placed in-office in the left eye under a bandage contact lens (−0.25 DS), with placement in the right eye planned for the one-week recheck. Amniotic membrane transplantation (AMT) was indicated for progressive keratitis with reduced corneal sensation refractory to lubrication and corticosteroids, to supply anti-inflammatory and neurotrophic matrix factors and promote epithelial healing, consistent with accepted management of early neurotrophic keratopathy (14). Topical cyclosporine 0.05% eyedrops were started twice daily, prednisolone was discontinued, and preservative-free artificial tears were continued four times daily.

At the one-week recheck, the left eye showed no noticeable SPK (vital stain not instilled) with signs assessed as greatly improved, and BCVA had improved in both eyes. The right eye, which had received no treatment beyond drops, remained stable with 1+ central SPK, and the planned amniotic allograft (Apollo, 12 mm, with a −0.25 DS bandage contact lens) was placed in the right eye at this visit.

At the following recheck one week later (two weeks after the left-eye graft), the bandage contact lenses were removed. The right eye showed trace SPK (a single pinpoint stain) with BCVA improved to 20/25. In the left eye, signs had partially recurred after removal of the membrane and bandage lens, with 1+ SPK in an inferior band, although vision was stable at 20/30. Preservative-free artificial tears four times daily and cyclosporine 0.05% twice daily were continued, a nightly lubricating gel/ointment was added in both eyes for long-term use, and a three-month follow-up was scheduled, with topical cenegermin (Oxervate, recombinant human nerve growth factor) to be considered at that visit if signs and symptoms had not improved.

Ribociclib was permanently discontinued at the seven-month oncology visit, after being held for two months, with the chart noting that rare ophthalmic side effects associated with ribociclib had been discussed and that, after weighing the risks and benefits, ribociclib would be discontinued. Anastrozole was continued.

### 2.4 Recovery and outcome

Vision improved after ribociclib withdrawal and with the amniotic membrane transplantation. The left eye’s early post-graft improvement partially receded after membrane and bandage-lens removal, and it remained the slower-recovering eye thereafter, consistent with the asymmetric severity at presentation. Five months after discontinuation, the right eye ocular surface had returned to baseline, and the left was improving. By nine months, she reported significant clinical improvement, with persistent mild left eye symptoms. At 15 months, both eyes had improved; she remained on anastrozole monotherapy and topical cyclosporine, with no recurrence of eye issues. The asymmetric recovery, faster in the less severely affected right eye, mirrored the asymmetric severity at presentation. At the Month 22 examination, the right cornea was clear without staining with TBUT greater than 10 seconds, the left eye showed trace SPK with TBUT of 7 seconds, and intraocular pressure was 19 mmHg in each eye; the assessment was mild, stable dry eye syndrome secondary to tear deficiency, with the ocular surface greatly improved compared with prior visits. Topical cyclosporine 0.05% twice daily and artificial tears four times daily were continued, with advice on lubrication during sustained computer use, and a routine six-month recall was given. The improvement in objective tear-film metrics (TBUT 6 seconds in each eye at the time of AMT versus greater than 10 seconds in the right eye and 7 seconds in the left eye at Month 22) parallels the symptomatic recovery. She adhered to and tolerated the topical regimen well, and no adverse or unanticipated events occurred during ophthalmic follow-up. Corneal sensation was re-assessed qualitatively at a follow-up visit and had recovered, and was noted as “normal”.

### 2.5 Concomitant medications and confounders

At the onset of ocular symptoms, the patient was also taking anastrozole 1 mg daily, fluoxetine 10 mg daily, fexofenadine 180 mg daily, alprazolam 0.25 mg nightly, omeprazole daily, melatonin nightly, and several vitamin supplements (per the oncology medication record). She worked in an office with approximately six hours of daily computer use, an additional ocular-surface stressor. All of these agents can affect the ocular surface; all were continued unchanged after ribociclib was stopped, and vision improved nonetheless. **Table 2** itemizes each agent, including dose, duration relative to ribociclib exposure, and known ocular-surface effects.

**Table 2:** Concomitant systemic medications at ocular symptom onset.

Medication (dose)	Frequency	Duration relative to ribociclib	Known ocular-surface effects
Anastrozole 1 mg	Daily	Started with abemaciclib (Month −2); continued throughout and after	Dry-eye signs/symptoms (aromatase inhibitor) (19)
Fluoxetine 10 mg	Daily	On board at symptom onset; continued throughout	Dry eye; mydriasis (SSRI class) (20, 21)
Fexofenadine 180 mg	Daily	On board at symptom onset; continued throughout	Anticholinergic drying (antihistamine class) (22, 23)
Alprazolam 0.25 mg	Nightly	On board at symptom onset; continued throughout	Possible mild drying (benzodiazepine class) (20)
Omeprazole 40mg	Daily	On board at symptom onset; continued throughout	None established
Melatonin 3mg	Nightly	On board at symptom onset; continued throughout	None established
Vitamin supplements (C, biotin-keratin, calcium, VitB12) Doses not recorded	Daily	On board at symptom onset; continued	None established

Doses and durations are per the oncology medication record. All agents were continued unchanged for more than 15 months after ribociclib discontinuation, during which vision progressively recovered.

### 2.6 Timeline

**Table 3** summarizes the case timeline, and **Table 1** summarizes standardized serial ocular findings.

**Table 3:** Timeline of clinical events.

Time point	Event
Month −37	Last comprehensive eye exam: no ocular surface disease, no corneal pathology
Month −13	Diagnosis: left invasive ductal carcinoma, grade 3, ER+/PR+/HER2−, cT2N1 (stage IIB)
Month −12 to −7	Neoadjuvant ddAC × 4 → weekly paclitaxel × 4
Month −6	Bilateral mastectomy + left ALND: pathologic complete response (ypT0, pN0(i+))
Month −4	Adjuvant radiation therapy completed (25 fractions)
Month −2	Adjuvant abemaciclib + anastrozole started; no vision complaints documented.
Month −1	Abemaciclib discontinued for refractory GI toxicity; bilateral salpingo-oophorectomy.
**Month 0**	**Ribociclib 400 mg/day (21/7 schedule) + anastrozole initiated**
Month 3	Oncology visit: explicitly denies vision changes; tolerating ribociclib. Later messages reported some concerns regarding dry eyes.
Month 4	First optometry visit: bilateral inferior SPK (1+ OD, 2+ OS); BCVA 20/20− OD, 20/20 OS with new prescription; preservative-free artificial tears (Refresh Digital PF) recommended
Early Month 5	Second optometry visit: corneal sensitivity graded 3+ central OU, 1+ superior OS, 0 all other quadrants; SPK migrated inferior → central (1+ OD, 2+ OS); TBUT 6 s OU; BCVA 20/25 + 2 OD, 20/30 OS; OD manifest refraction shifted −4.25 → −5.00 sphere over 30 days; assessment: possibly early neurotrophic; prednisolone acetate 1% four times per day started; documented next-line plan: AMT + cyclosporine
Mid-Month 5	**Ribociclib was held after no improvement in visual symptoms.**
Month 6	Third optometry visit: SPK now central, 1+ OD and 2+ OS; TBUT 6 s OU; IOP 21 mmHg OU; VA further reduced; no improvement after two weeks of corticosteroid; assessment “possibly early neurotrophic”; amniotic membrane transplantation, staged: Apollo Allograft 12 mm + bandage lens placed OS, with OD placement at one-week recheck; cyclosporine 0.05% twice daily started; prednisolone discontinued
Month 6 (+1 wk)*	One-week recheck: OS signs greatly improved, no noticeable SPK (stain not instilled); OD stable, 1+ central SPK; BCVA improved OU; OD amniotic allograft (Apollo 12 mm + bandage lens) placed; artificial tears QID and cyclosporine BID continued long-term
Month 6 (+2 wk)	Two-week recheck: bandage lenses removed; OD trace SPK (single pinpoint stain), BCVA 20/25; OS signs partially recurred after membrane/lens removal (1+ inferior band SPK), vision stable 20/30; nightly lubricating gel added OU; RTC 3 months, cenegermin (Oxervate) to be considered if not improved
Month 7	Ribociclib permanently discontinued; anastrozole continued.
Month 12*	Right eye vision normalized; left eye improving.
Month 16*	Continued bilateral improvement; left eye still mildly symptomatic
Month 22*	Bilateral vision improved; on anastrozole + cyclosporine; no evidence of disease. Exam: OD cornea clear, TBUT >10 s; OS trace SPK, TBUT 7 s; IOP 19 mmHg OU; mild stable dry eye.

AMT, amniotic membrane transplantation; ALND, axillary lymph node dissection; ddAC, dose-dense doxorubicin/cyclophosphamide; SPK, superficial punctate keratitis; BCVA, best-corrected visual acuity; OD, right eye; OS, left eye; OU, both eyes; RTC, return to clinic; BCL, bandage contact lens; QID, four times daily; BID, twice daily.

*Formal Snellen best-corrected visual acuity was not recorded at this encounter; visual status reflects the clinician’s documented qualitative assessment and the patient’s reported symptoms. The nearest documented Snellen measurements are those at the Month 6 + 2-week recheck (20/25 OD; 20/30 OS) and are shown in **Table 1**.

Bold indicates initiation and cessation of ribociclib.

## 3 Discussion

This case suggests three contributions to the ocular safety literature on CDK4/6 inhibitors. First, to our knowledge, it appears to be the first report of clinically assessed neurotrophic features, including reduced corneal sensitivity bilaterally, in a setting of progressive SPK refractory to standard therapy, occurring during ribociclib exposure. Previously reported ribociclib-related corneal events include vortex keratopathy (7–9) and epithelial keratopathy with punctate staining (10), consistent with lysosomal or epithelial injury rather than denervation. Second, the severity prompted a procedural intervention; to our knowledge, no prior ocular adverse event attributed to ribociclib has been managed with amniotic membrane transplantation. Third, it illustrates the value of prompt oncologic action, including holding the drug when ocular symptoms persisted despite standard topical therapy.

### 3.1 Causality assessment

Application of the Naranjo Adverse Drug Reaction Probability Scale (24, 25) yields a score of 5, in the probable range (5–8) (**Table 4**). Four positive findings anchor the score: ribociclib has prior published associations with corneal toxicity (7–9). The event appeared after the drug was started, with vision explicitly normal at the three-month oncology visit and SPK present at four months; the reaction improved after dechallenge; and the findings were objectively confirmed (slit-lamp SPK grading, serial visual acuity, and clinically assessed quadrant-graded bilaterally reduced corneal sensitivity).

**Table 4:** Naranjo adverse drug reaction probability scale.

#	Question	Answer	Score
1	Are there previous conclusive reports of this reaction?	Yes	+1
2	Did the adverse event appear after the suspected drug was administered?	Yes	+2
3	Did the reaction improve when the drug was discontinued?	Yes	+1
4	Did the reaction reappear when the drug was readministered?	Not done	0
5	Are there alternative causes that could, on their own, have caused the reaction?	Do not know	0
6	Did the reaction reappear when a placebo was given?	Not done	0
7	Was the drug detected in blood or other fluids in toxic concentrations?	Not done	0
8	Was the reaction more severe when the dose was increased, or less severe when decreased?	Unknown	0
9	Did the patient have a similar reaction to the same or similar drugs in any previous exposure?	No	0
10	Was the adverse event confirmed by any objective evidence?	Yes	+1
	**Total**	**Probable**	**5**

A total score of 5 corresponds to a probable adverse drug reaction (probable range, 5–8).

Bold indicates total Naranjo score.

The most consequential scoring decision concerns Question 5 (alternative causes). Given the presence of surgical menopause, aromatase-inhibitor therapy, occupational screen exposure, and several medications with ocular-drying potential, alternative or contributory causes cannot be excluded with certainty; Question 5 is therefore conservatively scored as “Do not know” (0). We note, as narrative context rather than as a basis for scoring: the patient had a documented normal comprehensive eye examination three years before ribociclib initiation while already taking fluoxetine, fexofenadine, and other agents on her regimen; she had no ocular complaints during the preceding month of abemaciclib exposure on the same concomitant medications (an exposure that may have been too brief to be informative). She remained on those medications and on anastrozole unchanged for more than 15 months after ribociclib discontinuation, during which time her vision progressively recovered with objective tear-film improvement (TBUT 6 s in each eye at the time of AMT versus >10 s and 7 s at Month 22). We did not perform rechallenge, which would have been ethically inadvisable; placebo and drug levels were not applicable.

We acknowledge that the Naranjo algorithm was not designed for complex oncology polypharmacy, that several items (rechallenge, placebo, drug levels, dose–response) are inapplicable or unavailable in routine care, and that inter-rater variability is well described (25). The score should therefore be interpreted as a structured framework supporting a probable association rather than as proof of causation.

### 3.2 Proposed mechanisms and biological plausibility

No mechanism was directly demonstrated in this patient; the published evidence summarized below is presented solely to establish biological plausibility for a proposed association. The cornea is a highly innervated tissue (12, 13). Trigeminal nerve fibers provide both sensory and trophic input that maintain the corneal epithelium, drive blink and tear reflexes, and support limbal stem cell function (12, 14). Three converging hypothetical mechanisms make ribociclib-induced corneal-nerve toxicity biologically plausible.

CDK4-dependent Schwann cell biology. Atanasoski and colleagues showed that postnatal Schwann cell proliferation is strictly and uniquely dependent on CDK4: neither CDK2 nor CDK6 can compensate, and after nerve injury, Schwann cells lacking CDK4 fail to re-enter the cell cycle (26). Schwann cells are essential for peripheral nerve maintenance and regeneration, including the production of neurotrophic factors (27, 28). Pharmacological CDK4 inhibition by ribociclib could therefore hypothetically impair the homeostatic turnover of Schwann cells supporting corneal nerves, producing slow attritional denervation rather than acute toxicity, which would be consistent with the four-month interval to onset observed here (29).

Limbal Schwann cells and epithelial renewal. More recently, limbal Schwann cells have been shown to directly regulate corneal epithelial renewal in mouse models: targeted ablation of corneal Schwann cells reproduces the phenotype of neurotrophic keratopathy, with epithelial breakdown and impaired wound healing (4). CDK4-dependent depletion of this Schwann-cell pool offers a hypothetical mechanistic bridge between systemic CDK4/6 inhibition and the ocular-surface phenotype observed in our patient, though this has not been demonstrated in humans.

Lysosomal sequestration in corneal epithelium. Llanos and colleagues demonstrated that all three clinically approved CDK4/6 inhibitors are sequestered into lysosomes through their weakly basic chemistry, with downstream effects on lysosomal biology (30). Subsequent FAERS-based work proposes that ribociclib lysosomal trapping in corneal epithelial cells is the mechanism underlying ribociclib-associated myopia and corneal opacities (6). Lysosomal injury could plausibly amplify Schwann-cell-driven trophic failure by additionally compromising the epithelial layer that depends on neurotrophic input, although this, too, remains conjectural.

These proposed mechanisms are not mutually exclusive. A unifying but speculative hypothesis is that ribociclib produces a combined insult to the corneal neurotrophic axis, which reduces CDK4-dependent Schwann cell turnover at the trigeminal end-organ, and simultaneously stresses the corneal epithelium through lysosomal sequestration, such that patients with additional risk factors for ocular surface disease (here, aromatase inhibitor use, post-oophorectomy estrogen depletion, anticholinergic comedications) cross the threshold to clinically apparent neurotrophic keratitis. None of these mechanisms has been experimentally demonstrated for ribociclib in the human cornea; dedicated experimental and pharmacovigilance studies would be required to evaluate this suggested link among CDK4, Schwann cells, and lysosomes.

### 3.3 Comparison with previously reported ocular events

We searched PubMed/MEDLINE from database inception through September 2026 using combinations of the terms “ribociclib,” “palbociclib,” “abemaciclib,” “CDK4/6 inhibitor,” “cornea,” “keratitis,” “keratopathy,” “neurotrophic,” and “amniotic membrane,” without language restriction. We could not identify any prior report of neurotrophic keratitis, reduced corneal sensation, or amniotic membrane transplantation associated with any CDK4/6 inhibitor. The reports identified are summarized below.

Saeed and colleagues described bilateral vortex keratopathy after 10 months of ribociclib, with fine, golden-brown, whorl-like deposits typical of cationic amphiphilic drug accumulation (8). A more recent report described bilateral epithelial keratopathy with punctate staining during the third cycle of ribociclib in an 83-year-old patient; ribociclib was discontinued, but the epithelial findings did not regress, and acuity did not recover during follow-up (10). Martos and colleagues reported two cases of palinopsia in metastatic patients on 600 mg/day ribociclib with letrozole; in both, neuro-ophthalmological evaluation and brain MRI were unremarkable, and dose reduction allowed continued therapy (11). The FAERS analysis by Zhang and colleagues identified 66 ocular adverse event signals across the CDK4/6 class, with myopia, cataract, and blindness among the strongest, and ribociclib carrying significantly higher ocular toxicity than abemaciclib on multivariate analysis (6).

Considering the class as a whole, a recent masked, controlled cross-sectional study of 132 women found punctate epitheliopathy in 44.4% of eyes receiving a CDK4/6 inhibitor with an aromatase inhibitor versus 4.7% with an aromatase inhibitor alone and 2.3% in untreated controls, with vortex keratopathy in 13.3% of the combination group and in no other group independent of aromatase-inhibitor exposure, treatment duration, and tear-film parameters (31). This supports a direct epithelial class effect. Notably, 77.8% of that study’s CDK4/6 inhibitor group received ribociclib, and its authors state that the findings most directly apply to this agent; corneal sensation was not assessed in that study (31). The palbociclib label lists increased lacrimation (6.8%), blurred vision (5.5%), and dry eye (4.4%) among ocular reactions (32), and in the FAERS analysis palbociclib exhibited distinct signals, including cataract (6); however, no published palbociclib case has, to our knowledge, described corneal denervation. For abemaciclib, epiphora (≈7%) is the principal labeled ocular event (6). Prior CDK4/6 inhibitor corneal reports share with the present case an epithelial localization and, in some instances, improvement on drug withdrawal; they differ in that none documented reduced corneal sensation, refractoriness to topical therapy, or the need for a procedural intervention.

The present case differs from each of the others in clinically meaningful ways. Within the scope of the search described above, it appears to be the first description of reduced corneal sensation, which can be strongly associated with neurotrophic disease, rather than vortex deposits or epithelial change alone; the first managed with amniotic membrane placement; and the first refractory to standard topical corticosteroid and lubrication. The same patient reported no ocular complaints during approximately one month of abemaciclib exposure. This exposure may, however, have been too brief to produce attritional corneal nerve toxicity, and no inference about a differential ocular safety profile between the two agents can be drawn from a single patient; the observation is consistent with, but cannot confirm, the FAERS-derived signal of greater ocular toxicity with ribociclib than abemaciclib (6).

### 3.4 Clinical implications

Adjuvant ribociclib is administered for up to three years and is now indicated in patients with stage II–III HR-positive/HER2-negative early breast cancer (1, 17). In NATALEE, approximately 20% of patients discontinued ribociclib due to adverse events, with a median time to discontinuation of approximately 4 months—closely paralleling the time to corneal symptom onset in this case (1). As exposure increases, low-frequency yet high-impact ocular events warrant heightened surveillance.

Her oncologist responded promptly to non-resolving ocular symptoms by holding ribociclib immediately. Definitive ophthalmic management followed. The gradual, painless visual decline characteristic of neurotrophic keratopathy is easily attributed to a need for new corrective spectacles or to mild dry eye, and a structured oncology review of systems may not reliably surface it. Routine, explicit questioning about visual symptoms and accessible communication pathways for between-visit symptom reporting, such as patient-initiated reporting through electronic health portals, telephone triage, or video visits, may serve as a meaningful safety net for adverse events of this kind.

Recognizing that a single case cannot support evidence-based practice recommendations, we offer the following as potential clinical approaches and points of awareness for prospective evaluation as adjuvant CDK4/6 inhibitor use expands:

- Consider asking explicitly about visual symptoms including blurred vision, dryness, foreign-body sensation, photophobia at every oncology visit during CDK4/6 inhibitor therapy, rather than relying on open-ended review of systems.
- A low threshold for optometry or ophthalmology referral, including formal corneal sensitivity testing (e.g., Cochet-Bonnet esthesiometry), may be warranted in patients with persistent visual symptoms not responding to topical therapy within 2–4 weeks.
- Baseline ophthalmologic assessment could be considered for patients with stacked ocular surface risk factors (concurrent aromatase inhibitor, post-menopausal status, anticholinergic medications, prior ocular surface disease).
- If neurotrophic features (reduced corneal sensation, SPK refractory to topical therapy) are identified, interruption or discontinuation of CDK4/6 inhibitors may be prudent, pending confirmation of this association, given the risk of progression to persistent epithelial defects and surgical morbidity.
- In patients intolerant of one CDK4/6 inhibitor, the choice of a second agent may reasonably weigh the pharmacovigilance-derived ocular safety signal between ribociclib and abemaciclib, recognizing that comparative safety cannot be inferred from single cases.
- In summary, painless, progressive blurred vision during CDK4/6 inhibitor therapy is easily misattributed to a refractive change. Persistent or worsening ocular symptoms that do not respond to lubrication within 2 to 4 weeks may warrant ophthalmic referral with assessment of corneal sensation, and consideration of interrupting the drug.

### 3.5 Confounders and limitations

We systematically considered recognized causes of neurotrophic keratopathy (14). Herpetic keratitis was considered unlikely on clinical grounds: no herpetic eye disease was documented, and no dendritiform lesions were observed at any examination. Diabetes mellitus was denied in the documented history, with lab work showing normal-range blood glucose. Injury of the eye region was denied, and no ocular surgery, corneal procedure, or neurosurgical intervention affecting the trigeminal pathway was reported or endorsed by the patient. The patient did not wear contact lenses before symptom onset and used no chronic prescribed topical medications. No autoimmune or neurologic disease had been diagnosed. The remaining candidate contributors are the hormonal factors and systemic medications discussed below.

Aromatase inhibitor use is associated with mild dry-eye signs and symptoms in patients with breast cancer (19). Post-oophorectomy estrogen depletion may further compromise the ocular surface (9). Several concomitant medications (fluoxetine, fexofenadine, alprazolam) have anticholinergic or drying properties (20, 21, 23). Occupational computer use of approximately six hours daily is a further recognized ocular-surface stressor (33). These factors are plausible permissive or contributory influences, and their contribution cannot be excluded; the longitudinal record, however, makes any of them unlikely to be a sufficient cause on its own: reduced corneal sensation, refractoriness to topical corticosteroids and cyclosporine, and the need for amniotic membrane transplantation exceed what is typical for aromatase-inhibitor-associated dry eye. The temporal pattern is also informative. The patient was 4 months post-oophorectomy, remained on anastrozole and all other medications when ribociclib was discontinued, and experienced improved vision. This sequence is most consistent with ribociclib as the dominant precipitant against a permissive ocular-surface background, while falling short of establishing it.

Because amniotic membrane transplantation and topical cyclosporine were introduced close to ribociclib withdrawal, the relative contributions of dechallenge and ophthalmic therapy to the observed recovery cannot be separated. The staged placement provides a partial within-patient contrast: one week after the left-eye allograft, the treated left eye showed no noticeable SPK while the as-yet-untreated right eye remained stable with 1+ central SPK, and the right eye in turn improved to trace SPK within one week of its own graft, suggesting that the membranes contributed materially to early epithelial recovery. That the left eye’s signs partially recurred after membrane and bandage-lens removal, and that full recovery of both eyes nonetheless followed over the ensuing months off ribociclib, is consistent with the membranes providing a transient reparative effect while drug withdrawal enabled durable healing. This interpretation, however, remains observational. The sustained, progressive improvement over more than 15 months without recurrence, while anastrozole and all other systemic medications were continued unchanged, suggests that ribociclib withdrawal was necessary for recovery, but the surgical and topical interventions plausibly accelerated epithelial healing.

As a single case, this report cannot establish causality. Rechallenge was not performed and would have been ethically inadvisable; dose reduction, which can provide additional within-patient evidence, was likewise not undertaken. Corneal sensitivity was assessed clinically (graded semi-quantitatively by quadrant, 0 to 3+) rather than quantified by esthesiometry; *in vivo* confocal microscopy of the sub-basal nerve plexus was not available, and anterior-segment optical coherence tomography and corneal topography were not performed. Slit-lamp photographs were not obtained as part of routine care at the treating community practice, so no pre- and post-treatment images are available. Ribociclib plasma exposure was not measured. Methods to measure ribociclib include liquid chromatography, tandem mass spectrometry in plasma and tissue, voltammetric and spectrophotometric assays, and characterization of its degradation products, though we did not have access to these techniques (34–37). Although the temporal pattern argues against the concomitant medications and aromatase inhibitor as primary drivers, their contribution as permissive or contributory factors cannot be excluded.

## 4 Conclusion

We describe what appears, to our knowledge, to be the first case of bilateral neurotrophic keratitis reported in association with a CDK4/6 inhibitor, occurring during adjuvant ribociclib for early breast cancer and requiring bilateral amniotic membrane transplantation and permanent drug discontinuation. A Naranjo score of 5 places this in the probable range, supported by a normal ocular baseline, the absence of toxicity during a brief prior abemaciclib exposure (recognizing that one month may have been too short an exposure) and a positive dechallenge with continued anastrozole. As a single, signal-generating case, this report cannot establish causality; additional case reports and pharmacovigilance analyses are needed for confirmation. The biologically plausible, but experimentally unproven, convergence of CDK4-dependent Schwann cell vulnerability, limbal Schwann cell-mediated epithelial renewal, and lysosomal sequestration of ribociclib in corneal epithelium provides a hypothetical mechanistic basis for the phenotype. These interpretations are constrained by the limitations inherent to a single case report: rechallenge was not performed for ethical reasons, no dose–response information is available, ribociclib plasma concentrations were not measured, corneal sensitivity was assessed qualitatively rather than by quantitative esthesiometry, and the sub-basal nerve plexus was not imaged by *in vivo* confocal microscopy. As adjuvant CDK4/6 inhibitor use expands, oncologists should ask actively about visual symptoms and refer early to ophthalmology when symptoms persist.

## 5 Patient perspective

The patient reported that her vision gradually changed over several weeks and that she initially attributed the symptoms to needing an updated lens prescription. After seeing her optometrist, she was told the ocular surface was the problem and was started on steroid and lubricant drops. After a month with no improvement and a sense that her vision might be worsening, she contacted her oncologist directly through the electronic health portal. Her oncology team advised her to hold ribociclib. She was urgently referred back to her eye doctor to escalate treatment. She expressed appreciation for the quick clinical response and the urgency of the subsequent intervention. She continues to follow up with her oncologist and noted that this has increased her trust in her oncology care. She gave full verbal and written permission to publish her case.

## Data Availability

The original contributions presented in the study are included in the article/supplementary material. Further inquiries can be directed to the corresponding author.
